# Agreement Between Clinically Measured Weight and Self-reported Weight Among Patients With Type 2 Diabetes Through an mHealth Lifestyle Coaching Program in Denmark: Secondary Analysis of a Randomized Controlled Trial

**DOI:** 10.2196/40739

**Published:** 2022-09-14

**Authors:** Albi Imeraj, Thomas Bastholm Olesen, Ditte Hjorth Laursen, Jens Søndergaard, Carl Joakim Brandt

**Affiliations:** 1 Research Unit for General Practice, Department of Public Health University of Southern Denmark Odense Denmark; 2 Steno Diabetes Center Odense Odense University Hospital Odense Denmark; 3 Department of Public Health University of Copenhagen Copenhagen Denmark

**Keywords:** telemedicine, digital behavioral coaching, lifestyle change, mobile intervention, obesity, diabetes, patient engagement, validation, self-report, body weight

## Abstract

**Background:**

Digital health interventions are increasingly used to handle and promote positive health behaviors. Clinical measures are often used, and a certain precision is essential for digital health interventions to have an effect. Only few studies have compared clinically measured weights with self-reported weights. No study has examined the validity of self-reported weight from a mobile app used in a tailored weight loss intervention.

**Objective:**

The aim of this study was to analyze the agreement between clinically measured weight and self-reported weight collected from a mobile health lifestyle coaching program during a 12-month weight loss intervention for obese patients with and without type 2 diabetes. The secondary aim was to investigate the determinants for possible discrepancies between clinically measured and self-reported weights of these patients with different demographic and lifestyle characteristics and achievements of weight loss goals.

**Methods:**

Weight registrations were collected from participants (N=104) in a Danish randomized controlled trial examining the effect of a digital lifestyle intervention on weight loss among obese patients with and without type 2 diabetes. Data were collected at baseline and after 6 and 12 months. Self-reported weight was measured at home and registered in the app.

**Results:**

Self-reported body weight was lower than the weight measured in the clinic after 6 months by 1.03 kg (95% CI 1.01-1.05; *P*<.001) and after 12 months also by 1.03 kg (95% CI 0.99-1.04; *P*<.001). After 6 months, baseline weight and BMI were associated with a discrepancy of 0.03 kg (95% CI 0.01-0.04; *P*=.01) and 0.09 kg (95% CI 0.02-0.17; *P*=.02) per increment of 1 kg and 1 kg/m^2^, respectively, between clinically measured weight and self-reported weight. Weight change during the first 6 months was also associated with a difference of 0.1 kg (95% CI 0.04-0.01; *P*<.001) per kilogram of difference in weight between clinically measured weight and self-reported weight. Participants who did not achieve the 5% weight loss goal underestimated their weight by 0.79 kg (95% CI 0.34-1.23) at 6 months. After 12 months, only baseline weight was associated with a discrepancy of 0.03 kg (95% CI 0.01-0.05; *P*=.02) per increment of kilogram between clinically measured weight and self-reported weight. None of the other factors showed any significant discrepancy after 12 months.

**Conclusions:**

Self-reported weight obtained from mobile health is a valid method for collecting anthropometric measurements.

**Trial Registration:**

ClinicalTrials.gov NCT03788915; https://clinicaltrials.gov/ct2/show/NCT03788915

## Introduction

Systematic reviews show that there are several digital health interventions (DHIs) currently that aim to handle and promote positive health behaviors, such as mobile health (mHealth) or web-based interventions [1-6]. DHIs can improve health behavior and weight loss at a reasonable cost [1-6]. Obesity is associated with chronic lifestyle diseases such as type 2 diabetes (T2D) mellitus, cardiovascular disease, and some forms of cancer [1,7]. This is especially the case for T2D, which is strongly correlated to weight gain and obesity. Pathophysiological studies [8,9] indicate that weight loss may normalize glucose control in approximately 50% of patients with T2D. Due to digital advancements, DHIs can now be used to handle and promote positive health behaviors, including self-reporting of weight to track weight loss. Despite this, no clear guidelines or infrastructure have yet been developed for how all these self-reported data should be handled and used in clinical practice. If digital solutions are to be useful, implementation and accessibility of self-reported data are essential. Self-reporting of weight loss is recommended as an effective weight loss strategy and can be performed via different types of DHIs [10]. DHIs are commonly used in both commercial programs and research studies [11,12]. So far, only few studies [13-15] have attempted to evaluate the validity of self-reported weight from web-based and paper-based programs against clinically measured weight, and these studies suggest that self-reported weight may be used as a valid, quick, and cost-effective alternative to clinically measured weight. Furthermore, few studies have reported that the validity of self-reported weight declines with increasing BMI and women tend to underestimate their own weight [13-15]. However, to our knowledge, no study has attempted to investigate the agreement between clinically measured weight and self-reported weight in a mobile app–based lifestyle coaching program. No study has examined whether clinically measured weight and self-reported weight differ (1) with achievement/nonachievement of own weight loss goals and (2) between follow-ups in a 12-month mHealth-based tailored weight loss intervention in a group of overweight people with and without diabetes, where correct weight control is essential.

The primary aim of this study was to determine the agreement between clinically measured weight and self-reported weight collected from an mHealth lifestyle coaching program (long-term Lifestyle change InterVention and mHealth Application [Liva]) during a tailored 12-month weight loss intervention for obese patients with and without T2D. The secondary aim was to investigate the determinants for possible discrepancies between self-reported and clinically measured weights of these patients with different demographic and lifestyle characteristics and achievements of weight loss goals.

## Methods

### Study Design

This study was a secondary analysis and examined the agreement between clinically measured weight and self-reported weight recorded in an mHealth-based solution among intervention participants (N=104) from an open randomized controlled trial (RCT). The control group in the RCT did not have access to the app and therefore had no self-reported weights. We excluded the control group for this study purpose. The RCT examined the effect of a digital lifestyle intervention on weight loss among obese patients with and without T2D. This analysis was conducted in 2 regions in Denmark: the Region of Southern Denmark with 22 municipalities and the Capital Region of Denmark with 28 municipalities. Data were collected from March 2019 to October 2021. All methods are described in further detail in the study protocol [16]. The self-reported weight was collected from the Liva Healthcare mHealth lifestyle coaching program. Patient data included in the study are pseudonymized. Participants granted their consent to make them available for research purposes. Consent was obtained explicitly in the sign-up flow before the use of the app/service.

### Ethics Approval

The RCT was approved by the scientific and ethics committee of the Region of Southern Denmark according to Danish law (approval 18803) and registered on clinicaltrials.gov (NCT03788915).

### Participants and Eligibility Criteria

In each municipality within the participating regions, the participants were recruited through general practitioners and local health centers, the Danish Diabetes Association, and advertisements via social media. The participants registered through the Liva Healthcare app [16]. After registration, a research assistant would contact the participant by phone to make sure that he/she met the following inclusion criteria: (1) BMI of 30-45 kg/m^2^, (2) diagnosed with T2D, and (3) age between 18 and 70 years. The following exclusion criteria were applied: (1) lack of internet access through computer or smartphone, (2) pregnancy or planned pregnancy, and (3) serious or life-threatening disease [16].

### Baseline Meeting and Follow-up Assessment

Participants gave written informed consent and informed the research assistant about their medications at the baseline meeting, and a brief medical examination of the participants was performed subsequently. The medical examination included measurements such as height (measured in centimeters without shoes), weight (without shoes and subtracted 1 kilogram for clothing), and waist and hip circumference (with tape measure around the waist). Weight was measured on a CE-marked high-quality calibrated scale from Tanita Corporation with a capacity of up to 270 kg and weight accuracy of 100 grams. The same measurements were taken at 6 and 12 months of clinical follow-up. As described in the study protocol [16], additional examinations were made but were not included in this study since they were not relevant to our objectives. However, these additional examinations could have an impact on adherence to the intervention.

### Data Collection of Self-reported Weight

**Figure 1:**
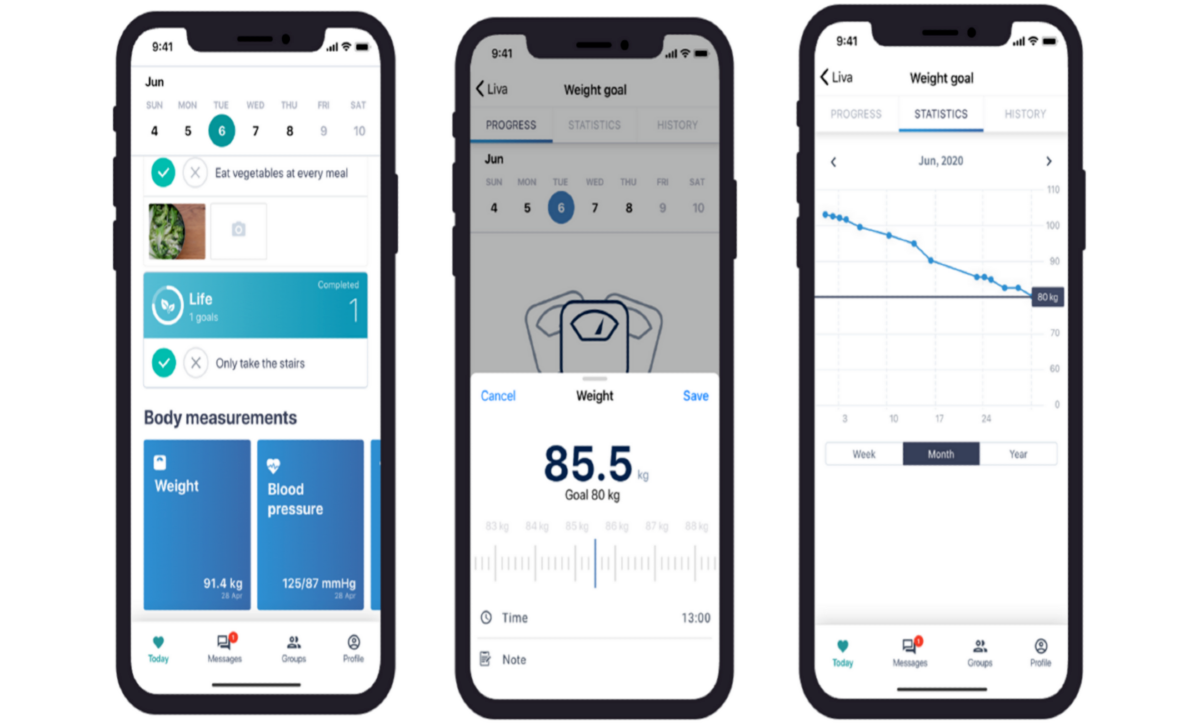
Screenshots of the Liva Healthcare app showing certain features, including weight measurement, tracking, and weight goals.

The intervention group received access to a lifestyle app/mHealth tool, where they received individual lifestyle coaching, completed daily tasks, and could send remarks or questions directly to the health care professionals (HCPs). The participants could set individual goals using the SMART (specific, measurable, attainable, relevant, timely) model [17], and based on these goals, the HCP could then provide weekly asynchronous digital coaching individualized for each participant. The HCP would inspire, commend goal attainment, and motivate the participant. Furthermore, the participants could register their own self-reported weight measured at home. Liva is built with the option to record and track individual weight every day, providing multiple measuring points. The Liva app also has an option to track data collected via Apple and Google Fitbit, as well as all other devices connected via Validic. The primary data that are imported are step data and daily activity. There were no specific requirements for their home measurements regarding calibration, type, etc. The participants were advised to always use the same scale to weigh themselves and were instructed that they should preferably do it on the same day of the week, for example, Sunday morning without clothes on (with underwear) but without shoes and after they had been to the toilet. This ensures the most uniform weight registration possible. The participants had to manually register their weights. Now, Liva offers synchronized bathing scales via an app so that data on weight, body composition, fat percentage, etc are measured and recorded automatically. But unfortunately, that was not a possibility when the Liva study was conducted and therefore, such parameters were not included. The program is also set up so that you receive notifications on your goals. If a participant has not registered a weight measurement on a certain day, he/she will receive a reminder. The mHealth tool is described in further detail in the Template of the Intervention Description and Replication [16]. As described earlier, the clinical weight measurements were taken at 6 and 12 months of clinical follow-up. To examine the agreement between these 2 measurement methods, we first had to define the limits for which self-reported weights could be used for the statistical analyses. As weight can change relatively quickly, the duration between the 2 measurements had to be reasonably close. To be included as a valid self-reported weight, the data point had to be 1-21 days prior to the 6 and 12 months of clinical follow-up. To minimize bias, we excluded self-reported weights on the same day or right after the clinical follow-up since our data showed that these self-reported weights were identical to the clinical measured weights (similar all the way down to decimals). This resulted in a total of 104 participants having a valid home measurement 1-21 days prior to the clinical assessments. The participants in this study did not know their clinical weights before registering their self-reported weights. Figure 1 shows screenshots of the Liva Healthcare app.

### HCPs in This Study

The digital lifestyle coaching was provided by an HCP through the mHealth tool. All the HCPs were educated as nurses, dietitians, physiotherapists, or occupational therapists. They all received special training on how to practice digital health coaching and had practiced it for at least 2 years. One primary HCP was assigned to each participant to achieve and secure a close and trusting professional relationship [16].

### Statistical Analysis

Differences in baseline characteristics between participants with and without valid home measurements were compared with analysis of variance for the continuous variables and chi-square test for the categorical variables. The following factors were included: gender, age, diabetes (yes/no), education, marital status, occupational status, baseline weight, and baseline BMI. Measured and self-reported weights were compared by linear regression (95% CI), and agreement was evaluated by Pearson correlation coefficients to determine the strength of the linear relationship. The degree of agreement between the self-reported and measured weight was also evaluated visually using Bland-Altman plots, and 95% limits of agreement were reported [18]. To identify determinants associated with the difference between measured and self-reported weights, we used linear regression and two-sided *t* test. Differences (clinical weight – self-reported weight) indicate if self-report was under (+) or over (–) estimated. Follow-up clinically measured weight was also used to determine the amount of weight change in participants classified as either achieving or not achieving the goal of 5% weight loss, which according to research is defined as a clinically relevant weight loss [19]. Mean values with corresponding standard deviations (SD) and frequencies with percentages have been reported. All analyses were performed using Stata version 13 (StataCorp LLC).

## Results

### Participant Characteristics

**Figure 2:**
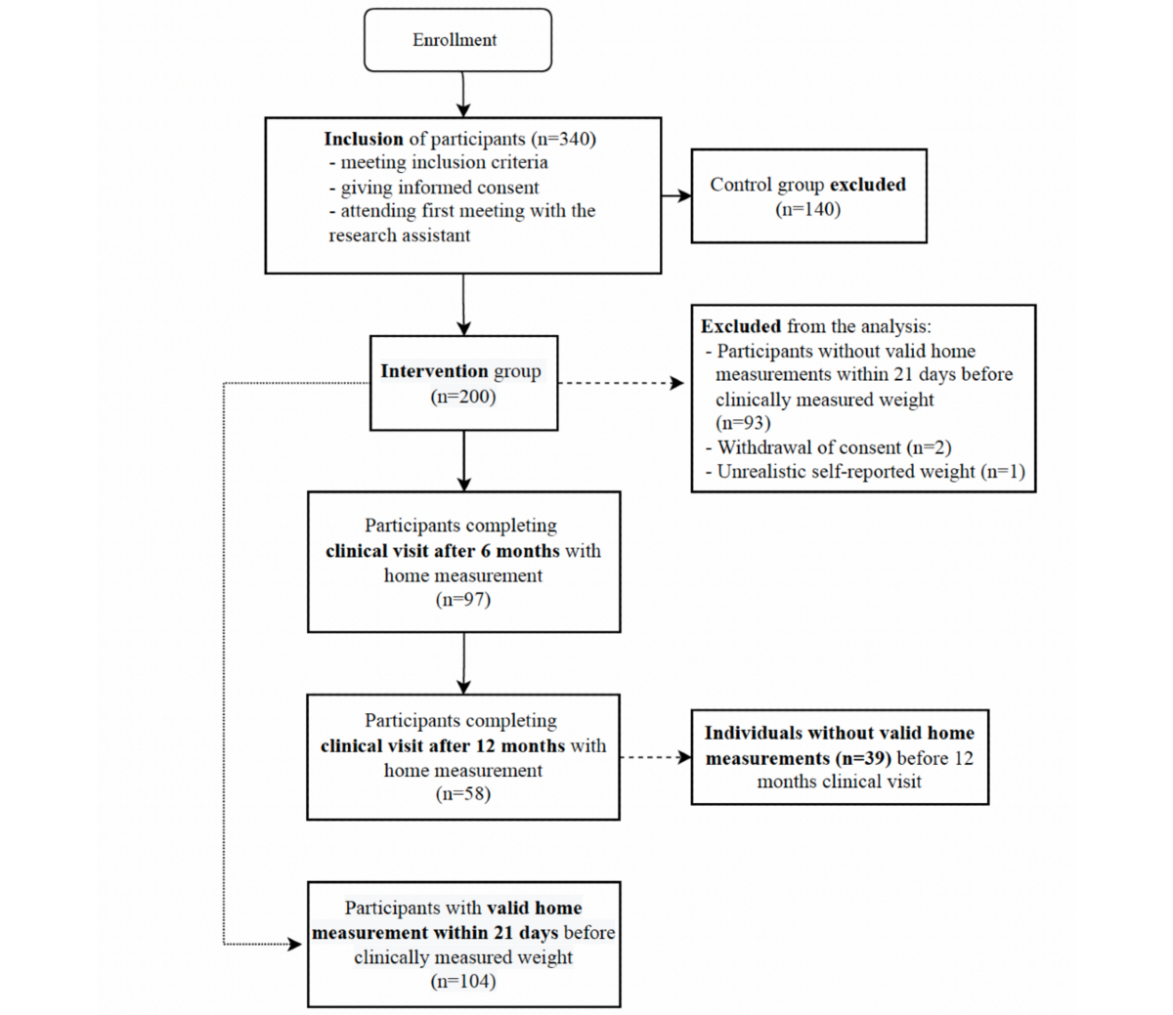
Participant flow during the study.

This study consisted of 200 participants from the intervention group of an RCT. Data were available after 6 months and 12 months, but 93 participants did not have a valid home measurement 1-21 days prior to their clinical weight measurement and were therefore excluded from the final analyses. Further, 3 participants were excluded because of withdrawal of consent and an unrealistic self-reported weight, with a 42-kg difference. As presented in Figure 2, the final sample consisted of 104 participants with a valid home measurement, of which 97 and 58 participants were present at the 6-month and 12-month follow-up, respectively. There were no demographic differences at baseline when divided into groups with and without a valid home measurement. Participants’ mean body weight was 103.9 kg, mean BMI was 35.3 kg/m^2^, and mean age was 52.1 years (Table 1). At 6 months and 12 months, 46 (44.2%) and 7 (6.7%) participants of the total 104 participants only had 1 self-reported weight, respectively, while 51 (49.1%) participants self-reported weights after both 6 and 12 months. Baseline characteristics of the participants with either 1 or 2 valid home measurements at 6-month and 12-month follow-ups did not differ in prevalence besides marital status. No differences in age, sex, disease, education, occupational status, and body composition were found (Multimedia Appendix 1). Furthermore, weight loss at 6 and 12 months did not differ between participants with and without a valid home measurement (data not shown).

Multimedia Appendix 2 shows the percentage distribution of the days between clinically measured weight and self-reported weights of the 104 participants within 1-21 days. At 6 months, 60 (57.6%) and 78 (75%) weight registrations were made within 7 and 13 days, respectively. At 12 months, 64 (61.5%) and 88 (84.6%) weight registrations were made within 7 and 13 days.

**Table 1:** Baseline characteristics of the participants in the study group without and with home measurements.

			Without home measurement		With home measurement		All		*P* value	
Participants, n (%)			96 (47.2)		104 (52.8)		200 (100)		N/A^a^	
Age (years), mean (SD)			51.8 (11.3)		52.4 (9.4)		52.1 (10.3)		.69	
**Sex, n (%)**										.31
	Female	57 (61.3)		71 (68.3)		128 (65)				
	Male	36 (38.7)		33 (31.7)		69 (35)				
**Diabetes, n (%)**										.43
	Yes	49 (52.7)		49 (47.1)		98 (49.7)				
	No	44 (47.3)		55 (52.9)		99 (50.3)				
**Education, n (%)**										.96
	None	14 (15.1)		14 (13.5)		28 (14.2)				
	Short (vocational courses, not university level)	23 (24.7)		26 (25)		49 (24.9)				
	Long (university level, bachelors and masters)	9 (9.7)		10 (9.6)		19 (9.6)				
	Middle (university level, bachelors)	45 (48.4)		53 (51)		98 (49.7)				
	Don’t know	2 (2.2)		1 (1)		3 (1.5)				
**Marital status, n (%)**										.17
	Married	55 (59.1)		77 (74)		132 (67)				
	Unmarried	23 (24.7)		16 (15.4)		39 (19.8)				
	Divorced	13 (14)		10 (9.6)		23 (11.7)				
	Widowed	2 (2.2)		1 (1)		3 (1.5)				
**Occupational status, n (%)**										.13
	Employed	62 (66.7)		79 (76)		141 (71.6)				
	Out of work (including on maternity leave or unemployment benefits)	8 (8.6)		8 (7.7)		16 (8.1)				
	Out of work (social benefits)	5 (5.4)		0 (0)		5 (2.5)				
	Early retirement	5 (5.4)		2 (1.9)		7 (3.6)				
	Retired	11 (11.8)		14 (13.5)		25 (12.7)				
	Student	2 (2.2)		1 (1)		3 (1.5)				
Weight (kg), mean (SD)			104.9 (17)		102.9 (14.3)		103.9 (15.6)		.36	
BMI, mean (SD)			35.7 (4)		34.9 (3.6)		35.3 (3.8)		.14	

^a^N/A: not applicable.

### Overall Difference Between Clinically Measured Weight and Self-reported Body Weight

**Figure 3:**
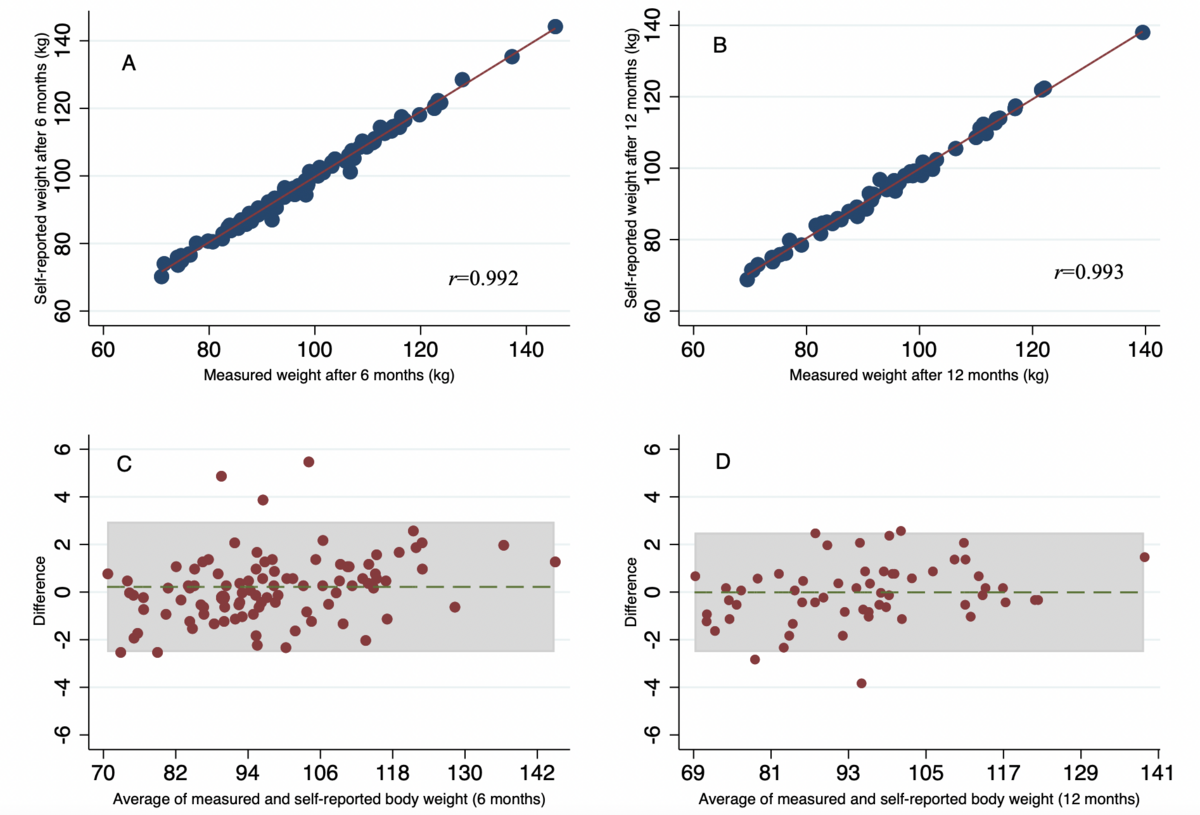
(A-B) Linear regression (95% CI) of measured and self-reported weights after 6 and 12 months of clinical follow-up. (C-D) Bland-Altman plot of difference between measured and self-reported weights (y-axis) in relation to the average of measured weight (x-axis) after 6 and 12 months of clinical follow-up. The solid area shows mean difference (2 SD) and the dashed line shows differences equal to zero. A negative sign in difference indicates overestimation. A positive sign indicates underestimation of self-reported weight.

The average difference between measured and self-reported body weights at the 6-month follow-up was 1.03 kg (95% CI 1.01-1.05; *P*<.001). The average difference between measured and self-reported body weights at the 12-month follow-up was also 1.03 kg (95% CI 0.99-1.04; *P*<.001). The Pearson correlation coefficient between measured and self-reported body weights showed a high correlation after 6 months (*r*=0.99) and 12 months of follow-up (*r*=0.99). The Bland-Altman plot showed a tendency of increased underestimation with greater clinically measured weight values (Figure 3).

### Possible Discrepancies and Predicting the Extent of Misreporting Among Participants

**Figure 4:**
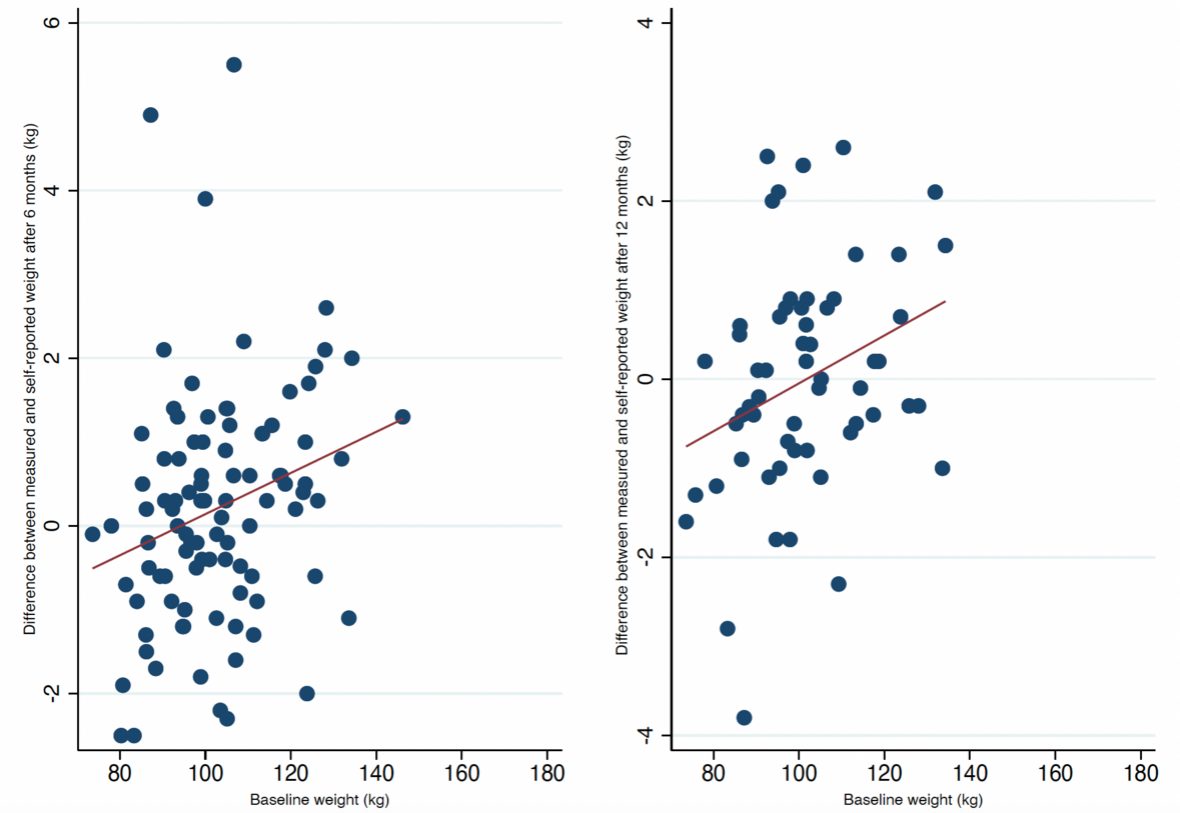
Scatter plots with fitted lines at 6 and 12 months of clinical follow-up grouped by baseline weight.

**Figure 5:**
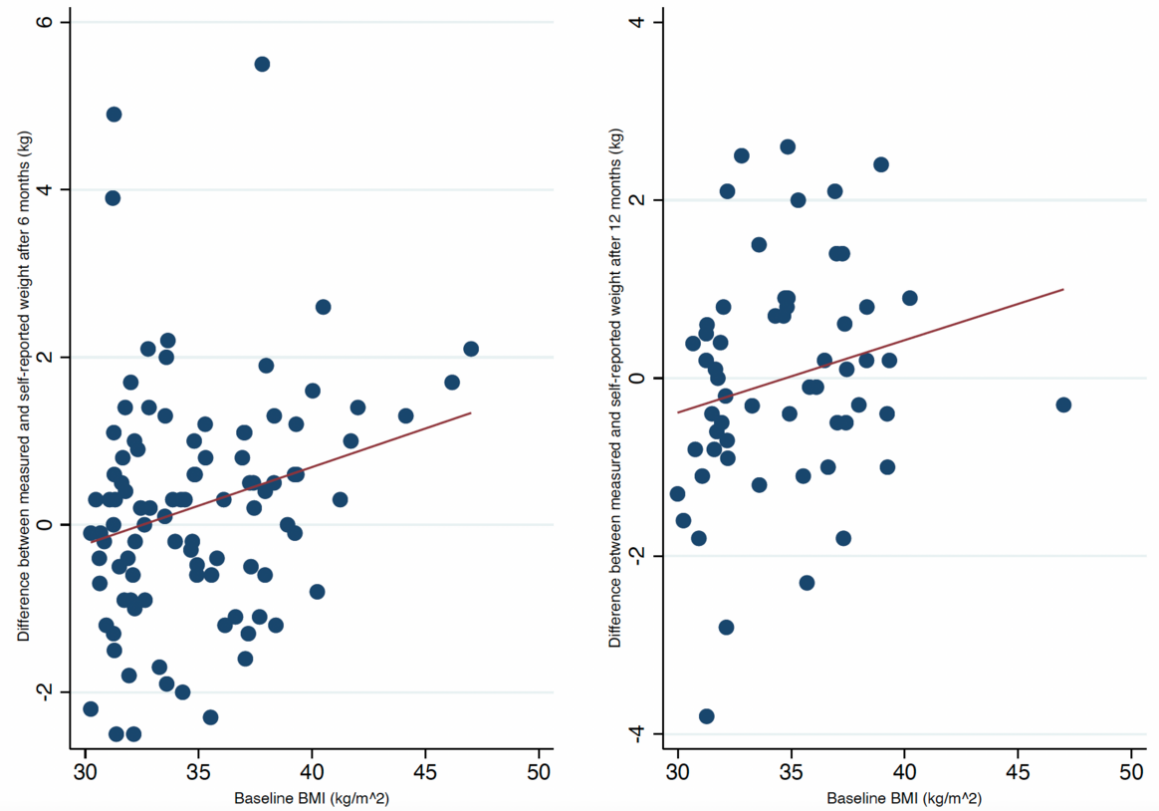
Scatter plots with fitted lines at 6 and 12 months of clinical follow-up grouped by baseline BMI.

**Figure 6:**
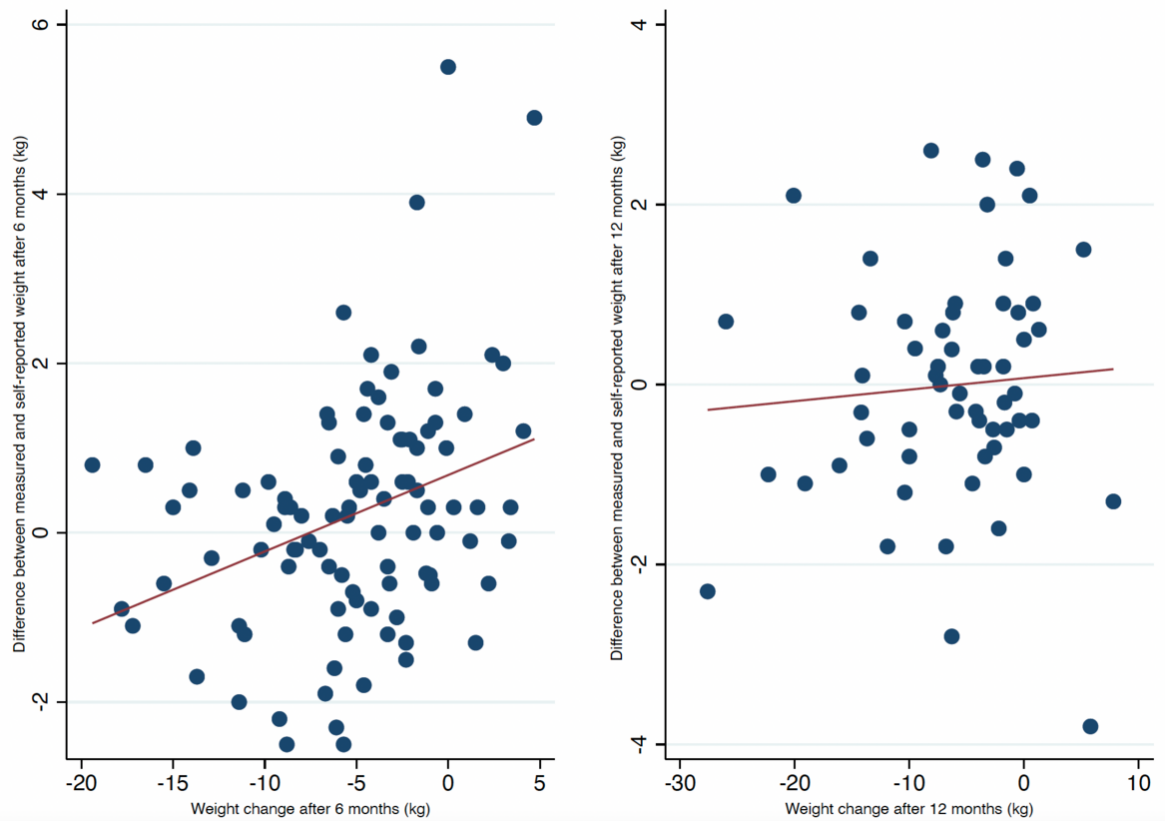
Scatter plots with fitted lines at 6 and 12 months of clinical follow-up grouped by weight changes during the intervention.

Baseline weight and BMI were associated with a discrepancy of 0.03 kg (95% CI 0.01-0.04; *P*=.01) and 0.09 kg (95% CI 0.02-0.17; *P*=.02) per increment of 1 kg and 1 kg/m^2^, respectively, between measured and self-reported weights (Figure 4 and Figure 5). Furthermore, weight change at 6 months was also associated with a difference of 0.1 kg (95% CI 0.04-0.01; *P*<.001) per kilogram of weight change between measured and self-reported weights (Figure 6). Achievement of the 5% weight loss goal was associated with a difference of –0.28 kg (95% CI –0.59 to –0.03) at 6 months. Those who did not achieve the 5% weight loss had a difference of 0.79 kg (95% CI 0.34-1.23), with a between-group difference of 1.08 kg (95% CI 0.54-1.60; *P*<.001) (Table 2). A within-group analysis was performed, and there were no significant differences between measured and self-reported weights when grouped by achievement/nonachievement of the 5% weight loss goal (Table 3). After 12 months, only baseline weight was associated with a discrepancy of 0.03 kg (95% CI 0.01-0.05; *P*=.02) per increment of kilogram between measured and self-reported body weights (Figure 4). Baseline BMI, weight change, and achievement of the 5% weight loss goal were not associated with discrepancies after 12 months. Educational status, marital status, employment status, and days between weight measurements were not associated with the differences in clinically measured and self-reported weights (not shown). Fewer participants self-reported their weight prior to the 12-month follow-up (Figure 2), but as shown in Multimedia Appendix 1, there were no significant differences from the baseline values of those who self-reported twice and once.

**Table 2:** Predicting discrepancy between clinically measured and self-reported weights by 5% weight loss goal attainment at 6 and 12 months of clinical follow-up (between-group difference).

Between-group difference	At 6 months (n=97)					At 12 months (n=58)			
	n	Mean (SE)	95% CI	*P* value	n		Mean (SE)	95% CI	*P* value
Achievement of 5% weight loss goal	56	–0.28 (0.15)	–0.59 to –0.03		32		–0.15 (0.20)	–0.58 to 0.26	
Did not achieve 5% weight loss goal	41	0.79 (0.22)	0.34 to 1.23		26		0.17 (0.27)	–0.39 to 0.73	
Difference		1.06 (0.26)	0.54 to 1.60	<.001			0.32 (0.34)	–0.35 to 1.00	.34

**Table 3:** Predicting discrepancy between clinically measured and self-reported weights by 5% weight loss goal attainment at 6 and 12 months of clinical follow-up (within-group difference).

Within-group difference	At 6 months (n=97)					At 12 months (n=58)			
	n	Mean (SE)	95% CI	*P* value	n		Mean (SE)	95% CI	*P* value
Achievement of 5% weight loss goal	56	0.22 (0.19)	–0.16 to 0.59	.25	32		0.03 (0.23)	–0.44 to 0.50	.90
Did not achieve 5% weight loss goal	41	0.22 (0.22)	–0.22 to 0.67	.33	26		–0.06 (0.25)	–0.57 to 0.45	.80

## Discussion

### Principal Findings

Our primary analysis showed a small, albeit statistically significant, average difference of 1.03 kg (*P*<.001) between measured and self-reported weights both after 6 and 12 months. The linear relationship between the clinically measured and self-reported weights was strongly correlated with high levels of agreement. Although the results show a significant difference, the magnitude of these differences was quite small, suggesting negligible clinical importance. Fluctuations in weight vary in the range of 0.5-1 kg per day [20-22], and a typical bathroom scale has an uncertainty of 1%-2% [23]. This supports the clinical validity of self-reported weight despite the modest discrepancy of 1.03 kg in our study. Moreover, participants maintained their reports over 12 months. Thus, self-reported mobile-based weights may be adequate and reliable for monitoring weight change during a coaching program. When grouped by different demographic and lifestyle factors, we found from our secondary analysis that participants with a higher baseline weight and BMI tended to underestimate their own weight by 0.03 kg and 0.09 kg at the 6-month follow-up, respectively. Furthermore, those who gained weight during the intervention also tended to underestimate their weight by 0.1 kg at the 6-month follow-up. Participants who achieved the 5% weight loss goal overestimated their weight by 0.28 kg. However, participants who did not achieve the 5% weight loss goal underestimated their weight by 0.79 kg at the 6-month follow-up. Although the within-group analysis did not show any differences, the between-group analysis indicated that those who achieved a 5% weight loss self-report more in accordance with their clinical weight. The discrepancies shown here were small, suggesting limited clinical relevance. None of the other demographic factors showed any significant discrepancies. Interestingly, the discrepancies improved over time when we analyzed the data from 6 to 12 months. As seen in the scatter plots, the data change from moving in a linear to a more constant pattern (closer to 0 in difference), which indicates that the previous discrepancy from baseline BMI and especially weight change improved from 6 to 12 months of clinical follow-up. This is also seen in the within-group analysis (Table 2), with an absolute change in difference of 0.22 kg to 0.03 kg in the achievement group and 0.22 kg to –0.06 kg in the nonachievement group, when we compare from 6 to 12 months (not significant). Furthermore, the between-group difference also improved by 1.06 kg to 0.32 kg from 6 to 12 months.

### Comparisons With Prior Research

Only few studies [13-15] have examined the agreement between measured and self-reported weights, with all of them being either paper-based or web-based through a web-based survey. To our knowledge, this is the first study to examine the agreement between clinically measured and self-reported weights from an mHealth-based lifestyle coaching program over 12 months. Our results show that the mobile-based reporting of own weight is a satisfactory method of data collection, which has also been proven in several international studies with web-based data collection [13-15,24]. Ekström et al [14] validated self-reported height, weight, and BMI among Swedish adolescents aged approximately 16 years by using a web-based survey. They found a mean difference of 1.1 kg between measured and self-reported weights, which was approximately the same as that found in our study (1.03 kg). Harvey-Berino et al [24] examined the agreement between measured and self-reported weights in a 6-month web-based obesity program. They found a mean underestimation of 0.86 kg. This overall positive agreement between self-reported and measured weight was further established in a nationwide cohort of 2643 US adults, which also found a relatively small underestimation [25]. According to our findings, baseline weight and BMI showed significant discrepancy, which agrees with several studies examining different populations [13-15,25]. Neermark et al [26] even found that calibrated values of self-reported BMI improved the predictive value of BMI for the risk of diabetes. Furthermore, a key finding from our study is that agreement between measured and self-reported weights appeared to worsen when participants gained weight and vice versa. Participants who were successful in losing weight between follow-ups reported a more accurate weight. Only few studies have examined weight change, but those that did, found the same results [24,27,28].

We found no other discrepancies in our data when participants were grouped by different demographic factors. This contrasts with other studies, which suggest that women tend to underestimate their own weight [13-15,25]. According to these studies, a possible explanation for this could be that women tend to be more aware of their own weight because of societal and psychological factors shaping their body image views. However, we found no statistical differences by gender. Koebnick et al [28] examined factors related to depressive symptoms among 17-year-old girls and found that strong experience of negative emotions such as anger, anxiety, and contempt was associated with underestimation of body weight. Furthermore, lower body satisfaction was associated with higher BMI, which led to higher negative emotions. Another key finding from our data indicates that a 12-month coaching intervention improves the discrepancy between measured and self-reported weights. Possible explanations for this could be that (1) the participants are regularly being observed and motivated by a coach with whom they have a good relationship between follow-ups, (2) they must measure their own weight at follow-up anyway, and (3) weight could be more stable after 12 months and thus, the difference of weight measured 21 days before the clinical visit is smaller. However, we cannot say whether this correlation implies causation. Our study design could not test whether being part of a coaching program improves the agreement between self-reported and clinically measured weights. In an optimal setting, there should have been a control group who did not receive coaching but still had access to the app and registered their weight. A previous study [29] demonstrated that regular feedback improved the validity of self-reported weight among obese employees. Contrary to the finding in our study, Jerome et al [27] found that the magnitude of underestimation doubled between 6 and 24 months of clinical follow-up. However, they also found that weight loss was associated with higher validity, which agrees with our and other studies. Jerome et al [27] also found that those with self-reported weight lost 3 times more weight compared to those without self-reported weight. This is important, because 39 (37.5%) of our 104 participants did not have a self-reported weight prior to the 12-month clinical follow-up. It can be assumed that lack of weight loss could have contributed to lesser motivation in this small group, resulting in fewer weight registrations. However, we did not find any differences regarding weight change between participants with and without self-reported weight. It is not quite clear whether weight loss encouraged accurate self-assessments or vice versa. Nevertheless, digital weight loss programs should be aware of this tendency.

Several differences in our study design might explain the different results. In our study, participants were older, more overweight, and had T2D, which could decrease the validity compared to populations with fewer overweight participants and with no chronic illnesses. Age could also impact the validity, because studies [30,31] show that younger populations have a higher usage of health apps and therefore are more prone to higher user engagement. Furthermore, we only chose self-reported weights 1-21 days prior to each clinical follow-up, with 65.3% (68/104) and 75% (78/104) of the participants self-reporting their weights within 7 and 13 days, respectively (Multimedia Appendix 2). This contrasts with that reported in other studies [14,15] where the limit of the self-reported measurements is further away from the date of the clinical measurements, although one study [27] had a limit of minimum 7 days. However, our data show that the number of days between measuring weights was not associated with discrepancy, including self-reported weights within 10 days. Jerome et al [27] also included self-reported weights from the same day as the clinical follow-up. We chose to exclude the self-reported weights that were made on the same day or right after the clinical measurements, since our data showed that many of the self-reported weights were identical to the clinically measured weights (similar all the way down to decimals). All the participants in our study were overweight or had T2D and were motivated to lose weight. This may limit the generalizability, since validity can vary with age, ethnicity, diseases, weight loss motivation, and several other variables. It is important to carry out validation studies in different countries and populations before generalization.

### Strengths and Limitations

The main strength of this study is the duration of the follow-up (12 months) compared to that in other studies, which made it possible to examine the agreement over time and assess whether a digital lifestyle coaching intervention had an impact on the validity. Our study sample was relatively large, taking into consideration that it was primary care–anchored and all the participants were overweight with T2D and motivated to lose weight. The participants were also not aware of this study, which could minimize potential bias. Clinical weight measurements were made according to a protocol by HCPs with a fair limit for days between self-reported and clinically measured weight.

The limitations in our study are as follows. In our study, first, only 58 (55.7%) out of 104 participants measured and self-reported weights at 12-month follow-up, which could induce a nonresponse bias and therefore limit the generalizability of our findings and affect the statistical power. However, baseline characteristics showed no differences (Multimedia Appendix 1), and weight change/loss was not significantly different between participants with and without self-reported weights. Second, we must also consider that weight fluctuations can happen during the day or during the menstrual cycle for females. However, from our analysis, gender did not influence any discrepancies. Lastly, clinical weight was measured with light clothes. Furthermore, the use of different measuring equipment by participants at home may have introduced measurement bias. We did not find any large systematic differences in the agreement between the clinically measured and self-reported weights.

### Conclusion

In this mHealth-based clinical trial, we found a high level of agreement between self-reported and clinically measured weights. Self-reported weight was, on average, underestimated by 1.03 kg. Baseline weight, baseline BMI, and weight change influenced this discrepancy. Our findings suggest that self-reported weights from an mHealth-based solution is a valid method for anthropometric measurements in a digital lifestyle intervention. Future studies should include longer follow-up periods with repeated measures over time.

## Appendix

Multimedia Appendix 1 Baseline characteristics of the participants with either 1 or 2 valid home measurements.

Multimedia Appendix 2 Percentage distribution of days between weights at 6 and 12 months of clinical follow-up.

## Abbreviations

DHI: digital health intervention
HCP: health care professional
Liva: Lifestyle change InterVention and mHealth Application
mHealth: mobile health
RCT: randomized controlled trial
T2D: type 2 diabetes
