# Supplementary material for: Agreement Between Clinically Measured Weight and Self-reported Weight Among Patients With Type 2 Diabetes Through an mHealth Lifestyle Coaching Program in Denmark: Secondary Analysis of a Randomized Controlled Trial
Source: JMIR Form Res. 2022 Sep 14;6(9):e40739. doi: 10.2196/40739 (PMC9520385; doi:10.2196/40739)
Supplement: Multimedia Appendix 1 [file formative_v6i9e40739_app1.pdf]

Supplementary table 1. Baseline characteristics of participants with either two or one valid home measurement.

	Only 6 month	Only 12 month	6 and 12 month	Total	P-value
n (%)	46 (44.2)	7 (6.7)	51 (49.0)	104 (100.0)	
Age, mean (sd)	51.4 (9.9)	54.3 (8.8)	53.1 (9.2)	52.4 (9.4)	0.60
Sex, n (%)					
Female	29 (63.0)	7 (100.0)	35 (68.6)	71 (68.3)	
Male	17 (37.0)	0 (0.0)	16 (31.4)	33 (31.7)	0.15
Diabetes, n (%)					
Yes	24 (52.2)	4 (57.1)	21 (41.2)	49 (47.1)	
No	22 (47.8)	3 (42.9)	30 (58.8)	55 (52.9)	0.48
Education, n (%)					
None	6 (13.0)	0 (0.0)	8 (15.7)	14 (13.5)	
Short	8 (17.4)	2 (28.6)	16 (31.4)	26 (25.0)	
Long	6 (13.0)	0 (0.0)	4 (7.8)	10 (9.6)	
Middle	26 (56.5)	5 (71.4)	22 (43.1)	53 (51.0)	
Don't know	0 (0.0)	0 (0.0)	1 (2.0)	1 (1.0)	0.54
Maritalstatus, n (%)					
Married	37 (80.4)	4 (57.1)	36 (70.6)	77 (74.0)	
Unmarried	5 (10.9)	1 (14.3)	10 (19.6)	16 (15.4)	
Divorced	4 (8.7)	1 (14.3)	5 (9.8)	10 (9.6)	
Widow	0 (0.0)	1 (14.3)	0 (0.0)	1 (1.0)	0.01
Occupationalstatus, n (%)					
Employed	33 (71.7)	6 (85.7)	40 (78.4)	79 (76.0)	
Out of work (including on maternary leave or unemployment benefits)	7 (15.2)	0 (0.0)	1 (2.0)	8 (7.7)	
Early retirement	0 (0.0)	0 (0.0)	2 (3.9)	2 (1.9)	
Retired	6 (13.0)	1 (14.3)	7 (13.7)	14 (13.5)	
Student	0 (0.0)	0 (0.0)	1 (2.0)	1 (1.0)	0.30
Weight (kg), mean (sd)	104.8 (13.8)	99.0 (10.6)	101.7 (15.1)	102.9 (14.3)	0.44
BMI, mean (sd)	35.2 (4.0)	34.9 (3.3)	34.6 (3.3)	34.9 (3.6)	0.68
