# Supplementary figures and images for: Agreement Between Clinically Measured Weight and Self-reported Weight Among Patients With Type 2 Diabetes Through an mHealth Lifestyle Coaching Program in Denmark: Secondary Analysis of a Randomized Controlled Trial
Source: JMIR Form Res. 2022 Sep 14;6(9):e40739. doi: 10.2196/40739 (PMC9520385; doi:10.2196/40739)

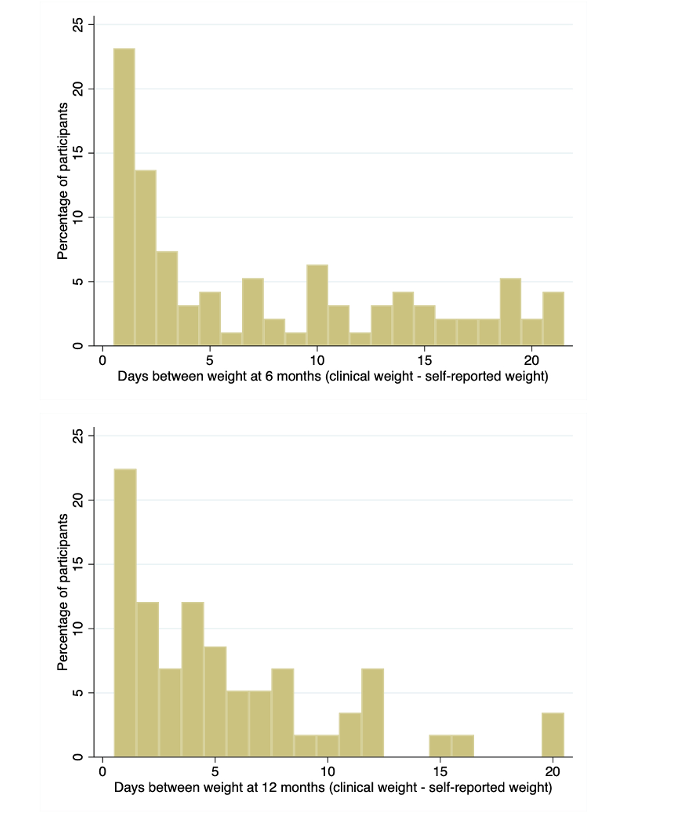

Supplement: Multimedia Appendix 2 [file formative_v6i9e40739_app2.png]
